# In-depth evaluation of false-positive biological reactions in the syphilis serological diagnosis: a cross-sectional study with two sampling components

**DOI:** 10.3389/fcimb.2026.1864213

**Published:** 2026-07-29

**Authors:** Jing Li, Xiaohan Li, Yike Huang, Fei Ye, Xi Lin, Dongdong Li

**Affiliations:** Department of Laboratory Medicine, West China Hospital of Sichuan University, Chengdu, China

**Keywords:** autoantibody, biological false-positive, syphilis, toluidine red unheated serum test, *Treponema pallidum*

## Abstract

**Objectives:**

This study aimed to systematically evaluate the disease spectrum, demographic characteristics, and autoantibody profiles associated with biological false-positive (BFP) reactions in serological tests for syphilis.

**Methods:**

A retrospective analysis was conducted on 87 patients with TRUST (+)/TPPA (-) results from January 2016 to February 2026 at West China Hospital, Sichuan University. Additionally, serum specimens from 1068 patients (including autoimmune diseases, Epstein-Barr virus infection, malignancy, and lupus anticoagulant positivity) were tested using chemiluminescence immunoassay and toluidine red unheated serum test (TRUST), and the autoantibody profiles of 539 of these patients were analyzed.

**Results:**

Among retrospective cases, unknown diseases accounted for the highest proportion (34.5%), followed by malignancy (12.6%) and neurological diseases (10.3%). In the cross-sectional case series, autoimmune diseases showed the highest BFP rate (52.3%), particularly rheumatoid arthritis (75.9%), connective tissue disease (62.4%), and Sjögren’s syndrome (52.3%); the false-positive rate was 36.1% in patients with malignancy and 8.5% in patients with lupus anticoagulant positivity. BFP titers were predominantly low (1:1 or 1:2). EBV-positive BFP reactions occurred only in the 1–19 years age group with acute infection. Autoantibody profiling revealed that, among BFP patients with autoimmune diseases, those who were ANA-positive (n=235) exhibited substantially higher positivity rates for U1-snRNP (21.7%), Sm (9.4%), SS-A (60.9%), SS-B (36.6%), CENP B (14.0%), ANuA (24.3%), AHA (15.3%), and ARPA (11.9%), whereas ANA-negative patients (n=28) showed only low positivity for SS-A (25.0%) and Scl-70 (7.1%).

**Conclusion:**

BFP reactions are most common in autoimmune diseases, particularly SLE, SS, CTD, and RA, and typically present with low TRUST titers. ANA-positive BFP patients exhibit a broad autoantibody profile, while ANA-negative patients show limited positivity. Comprehensive clinical and autoantibody evaluation is essential to avoid misdiagnosis and unnecessary treatment.

## 1 Introduction

Syphilis is a chronic, systemic sexually transmitted infection caused by *Treponema pallidum* (TP) subspecies, characterized by complex clinical manifestations and the ability to invade multiple organs and systems in the human body (Hook, 2017). Serological testing for syphilis includes treponemal and nontreponemal serological tests. Treponemal tests (TTs) mainly include *Treponema pallidum* chemiluminescence immunoassay (TP-CLIA) and *Treponema pallidum* particle agglutination assay (TPPA). TP-CLIA is a rapid, high-throughput automated detection method that utilizes paramagnetic microbeads coated with recombinant Tp15, Tp17, and Tp47 antigens to capture TP antibodies in serum (Satyaputra et al., 2021). TPPA, in contrast, uses purified TP organisms coated onto the surface of gelatin particles to prepare sensitized particles, which react with TP antibodies in serum, resulting in visible particle agglutination (Cao et al., 2023). Nontreponemal tests (NTTs), including the venereal disease research laboratory (VDRL) test, the rapid plasma reagin test (RPR), and the toluidine red unheated serum test (TRUST). NTTs are designed to detect immunoglobulin M (IgM) and IgG antibodies directed against lipoidal material and lipoidal protein-like substances released from damaged host cells, as well as cardiolipin potentially released by TP (Larsen et al., 1995; Gao et al., 2018). NTTs, which serves as a screening test for syphilis infection, can be used clinically to evaluate treatment response and to identify relapse or reinfection (Workowski et al., 2021).

A discordant syphilis test result is defined as a reactive NTT combined with a non-reactive TT; a finding commonly termed a biological false positive (BFP) result (Matthias et al., 2019). Due to the lack of specificity inherent to the TRUST assay, anti-lipoidal antibodies may also arise in non-syphilitic states, resulting in a BFP reaction despite the absence of active syphilis infection (Catterall, 1972). BFPs are closely associated with a diverse array of underlying diseases and physiological conditions, and their mechanisms are complex. These include autoimmune diseases (ADs) such as systemic lupus erythematosus (SLE) (Ahn et al., 2019) and antiphospholipid syndrome (APS) (Moore, 1952), acute or chronic infections including human immunodeficiency virus infection (Rompalo et al., 1992), Epstein-Barr virus (EBV) (Matsuura et al., 2025) and hepatitis C virus infection (Augenbraun et al., 2010), malignancies (Delaney, 1976), and recent vaccination (Monath and Frey, 2009; Korentzelos et al., 2022).

Clinically, BFP reactions present a daily diagnostic challenge. When a patient with no syphilis history shows positive TRUST but negative TP-CLIA, clinicians must navigate multiple possible etiologies—yet no data allow direct comparison of BFP risk across autoimmune, malignant, or infectious conditions. In pediatric practice, acute febrile illnesses with positive TRUST often trigger unnecessary antibiotic treatment, but whether age and infection phase modify BFP risk remains unanswered. Furthermore, when autoantibody testing is pursued, there is no evidence on whether comprehensive panels are warranted for all patients or should be stratified. These uncertainties not only risk misdiagnosis and wasted resources but also may delay appropriate management of underlying diseases, but may also impose unnecessary psychological distress, social stigma, and shame on patients. To address these specific dilemmas, this study systematically evaluated the disease spectrum, demographic characteristics, and autoantibody profiles associated with BFP reactions, aiming to provide clinically actionable guidance for real-world differential diagnosis.

## 2 Materials and methods

### 2.1 Study design and clinical sample collection

All syphilis serological test results from January 2016 to February 2026 were retrieved from the His database of the Department of Laboratory Medicine, West China Hospital, Sichuan University. A retrospective observational analysis was conducted on 87 patients with TRUST (+)/TPPA (-) results, and their clinical medical records were further reviewed. Additionally, between December 2025 and February 2026, residual serum samples were collected from 1,084 patients to establish a cross-sectional cohort that included patients with SLE, Sjögren’s syndrome (SS), connective tissue disease (CTD), rheumatoid arthritis (RA), EBV antibody positive, malignancies, and lupus anticoagulant (LAC) positivity. 1,084 samples were concurrently tested using the TP-CLIA and the TRUST. After excluding 16 TP-CLIA-positive samples due to confirmed or clinically suspected active/past syphilis infection, which would confound the identification of biological false-positive reactions, a total of 1,068 cases were included in the analysis, of which 539 underwent further autoantibody profile testing.

### 2.2 Laboratory test and reagents

The nontreponemal screening test was performed using the TRUST kit (Shanghai Rongsheng Biopharmaceutical Co., Ltd.) in accordance with the manufacturer’s instructions. For the qualitative test, 50 μL each of the positive control, negative control, and test serum was placed and evenly spread into the respective circles on the test card. One drop of TRUST reagent was then added vertically using the dedicated dropper. After shaking at 100 rpm for 8 minutes, the results were interpreted visually. For the quantitative test, the test serum was serially twofold diluted in normal saline, and the test was performed as described above. The highest dilution showing definite agglutination was taken as the agglutination titer of the serum. The treponemal antibody test was performed using the Roche e801 chemiluminescence immunoassay analyzer (F. Hoffmann-La Roche, Ltd.). The autoantibody profile, including U1 small nuclear ribonucleoprotein antibody (U1-snRNP), smith antibody (Sm), Sjogren’s syndrome type A antibody (SS-A), Sjogren’s syndrome type B antibody (SS-B), scleroderma-70 antibody (Scl-70), polymyositis-scleroderma antibody (PM-Scl), anti-histidyl-tRNA synthetase antibody (Jo-1), centromere protein B antibody (CENP B), proliferating cell nuclear antigen antibody (PCNA), anti-nucleosome antibody (ANuA), anti-histone antibody (AHA), and anti-ribosomal P protein antibody (ARPA), was measured using automated immunoassays based on “digital liquid-phase chip multiplex” technology. (Livzon Pharmaceutical Group Inc.). Antinuclear antibody (ANA) was determined using a fully automated indirect immunofluorescence instrument (EUROIMMUN Medical Diagnostics Co., Ltd.).

### 2.3 Statistical analysis

For statistical analysis and graphical representation, SPSS version 27.0 (IBM, USA) and GraphPad Prism version 10 (GraphPad, USA) were used. Descriptive statistics were applied to the main study variables, including disease type, TRUST titers, and autoantibody profiles, and results were expressed as absolute numbers (N) and relative frequencies (%). Appropriate statistical methods (chi-square test or Fisher’s exact test) were applied for intergroup comparisons based on the characteristics of the variables and the distribution across groups. A *P* value of < 0.05 was considered statistically significant.

## 3 Results

### 3.1 Retrospective patient characteristics

In this study, a total of 87 patients with BFP reaction were enrolled, and the distribution of disease spectrum is shown in **Table 1**. The disease types included malignancies, ADs, infectious diseases, cardiovascular diseases, and neurological disorders. Among them, patients without a definitive clinical diagnosis (classified here as ‘unknown’) were the most common, accounting for 30 cases (34.5%), which represented the largest proportion among all the distinct disease categories. Most of these patients were outpatients with a medical order for a syphilis test and no clinical data in the records. The remaining major disease categories included malignancy in 11 cases (12.6%), neurological diseases in 9 cases (10.3%), cardiovascular diseases in 8 cases (9.2%), and ADs in 6 cases (6.9%). The titers were predominantly at low levels: 34 cases (39.1%) had a titer of 1:1, 28 cases (32.2%) had 1:2, 13 cases (14.9%) had 1:4, 8 cases (9.2%) had 1:8, 3 cases (3.4%) had 1:32, and 1 case (1.1%) had 1:128. Overall, the vast majority of patients (86.2%) had TRUST titers ≤1:4, while only a small proportion (4.5%) had titers ≥1:32.

**Table 1:** Disease spectrum in 87 patients with BFP reaction (2016-2026).

Types of diseases	N	(%)
Malignancy	11	(12.6)
Autoimmune diseases	6	(6.9)
Infectious diseases	4	(4.6)
Respiratory diseases	5	(5.7)
Cardiovascular diseases	8	(9.2)
Neurological diseases	9	(10.3)
Urologic diseases	4	(4.6)
Musculoskeletal diseases	3	(3.4)
Endocrine diseases	1	(1.1)
Other	6	(6.9)
Unknown	30	(34.5)
Total	87	(100.0)

### 3.2 Age and sex stratified by BFP reaction

Excluding patients with positive treponemal antibodies, a total of 1068 patients were enrolled in this study, including 461 with BFP reaction and 607 without. Among patients with ADs, the proportions of those with and without BFP reaction were 52.3% and 47.7%, respectively. No statistically significant differences were observed between the two groups in age distribution (*P* = 0.242) or sex composition (*P* = 0.843), and both groups were predominantly female. Among patients positive for EBV antibodies, only 2 cases (2.2%) exhibited BFP reactions, both distributed in the 1–19 years age group and both positive for IgM antibodies, whereas no BFP reactions were observed in the 24–88 years age group. The difference in age distribution between the two groups was statistically significant (*P* = 0.023), while sex composition did not differ significantly (*P* = 0.950). Among patients with malignancy, those with BFP reaction accounted for 36.1% and those without, 63.9%. No statistically significant differences were found between the two groups in age distribution (*P* = 0.733) or sex composition (*P* = 0.052). Among patients positive for LAC, 7 cases (8.5%) were in the BFP reaction group. No significant difference in age distribution was observed (*P* = 0.782), whereas sex composition differed significantly (*P* = 0.048), with a higher proportion of females in the BFP reaction group (**Table 2**).

**Table 2:** Age (in years) and sex (Male/Female) of 1068 patients with or without BFP reaction.

Items	Total	Patients with BFP reaction (N = 461)		Patients without BFP reaction (N = 607)		
	N	N	(%)	N	(%)	P value
Autoimmune diseases	797	417	(52.3)	380	(47.7)	
≤39	320	166	(39.8)	154	(40.8)	0.242
40-59	324	179	(42.9)	145	(38.2)	
≥60	153	72	(17.3)	81	(21.3)	
Male/Female	73/724	39/378	(9.4)/(90.6)	34/346	(8.9)/(91.1)	0.843
EBV Ab (+)	92	2	(2.2)	90	(97.8)	
1-19	26	2	(2.2)	24	(26.1)	0.023
24-88	66	0	(0.0)	66	(71.7)	
Male/Female	48/44	1/1	(1.1)/(1.1)	47/43	(51.1)/(46.7)	0.950
Malignancy	97	35	(36.1)	62	(63.9)	
≤39	4	2	(5.7)	2	(3.2)	0.733
40-59	40	13	(37.1)	27	(43.5)	
≥60	53	20	(57.1)	33	(53.2)	
Male/Female	51/46	23/12	(23.7)/(12.4)	28/34	(28.9)/(35.1)	0.052
LAC (+)	82	7	(8.5)	75	(91.5)	
≤39	36	3	(42.9)	33	(44.0)	0.782
40-59	19	1	(14.3)	18	(24.0)	
≥60	27	3	(42.9)	24	(32.0)	
Male/Female	41/41	1/6	(1.2)/(7.3)	40/35	(48.8)/(42.7)	0.048

Significance notation: ns, P > 0.05 (not significant); *P ≤ 0.05; **P ≤ 0.01; ***P ≤ 0.001; ****P ≤ 0.0001.

### 3.3 BFP reactions and TRUST titers stratified by disease type

**Figure 1:**
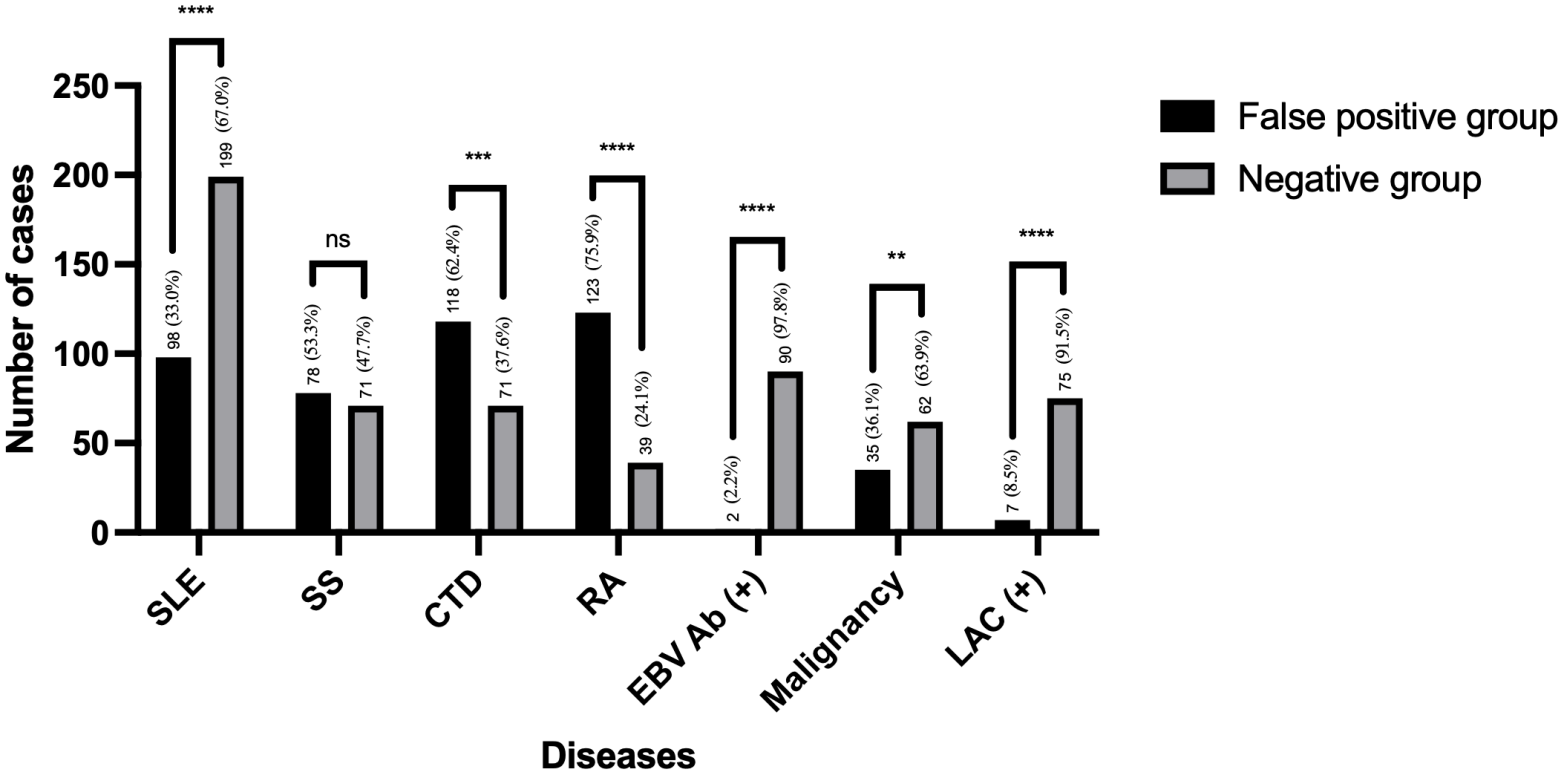
Distribution of the false-positive group and the negative group by disease.

The false-positive rate of the TRUST test was relatively high in patients with ADs, with low titers predominating. Among 797 patients with ADs, 380 (47.7%) were in the negative group, and 417 (52.3%) were in the false-positive group, of whom 351 (44.0%) had a titer of 1:1, 60 (7.5%) had a titer of 1:2, and 6 (0.8%) had a titer of 1:4. Specifically, the false-positive rate in patients with SLE was 33.0%, with titers mainly distributed at 1:1 (27.3%) and 1:2 (4.4%); in patients with SS, the false-positive rate was 52.3%, predominantly at 1:1 (34.2%) and 1:2 (18.1%); in patients with CTD, the false-positive rate was 62.4%, with 1:1 (54.5%) being the most common; in patients with RA, the false-positive rate was as high as 75.9%, of which 71.6% had a titer of 1:1 (**Table 3**). In contrast, the false-positive rate was only 2.2% in patients with positive EBV antibodies, 36.1% in patients with malignancy, and 8.5% in patients with LAC-positive (**Figure 1**). Moreover, false-positive titers in the above-mentioned populations did not exceed 1:2. Among the studied populations, patients with ADs showed a higher false-positive rate of the TRUST test, and false-positive titers were predominantly in the low-titer range.

**Table 3:** Distribution of TRUST titer in patients with and without BFP reaction.

Types of diseases	Total	Negative		1:1		1:2		1:4	
	N	N	(%)	N	(%)	N	(%)	N	(%)
ADs	797	380	(47.7)	351	(44.0)	60	(7.5)	6	(0.8)
SLE	297	199	(67.0)	81	(27.3)	13	(4.4)	4	(1.3)
SS	149	71	(47.7)	51	(34.2)	27	(18.1)	0	(0.0)
CTD	189	71	(37.6)	103	(54.5)	14	(7.4)	1	(0.5)
RA	162	39	(24.1)	116	(71.6)	6	(3.7)	1	(0.6)
EBV Ab (+)	92	90	(97.8)	2	(2.2)	0	(0.0)	0	(0.0)
Malignancy	97	62	(63.9)	35	(36.1)	0	(0.0)	0	(0.0)
Pulmonary	26	18	(29.0)	8	(22.9)	0	(0.0)	0	(0.0)
Gastrointestinal	38	21	(33.9)	17	(48.6)	0	(0.0)	0	(0.0)
Reproductive	33	23	(37.1)	10	(28.6)	0	(0.0)	0	(0.0)
LAC (+)	82	75	(91.5)	5	(6.1)	2	(2.4)	0	(0.0)

### 3.4 Distribution of autoantibodies in patients with BFP reactions

Among patients with ADs who developed BFP reactions, the distribution of autoantibody profiles was further analyzed, stratified by ANA status (**Table 4**). In the 235 ANA-positive BFP patients, variable positivity rates were observed across different autoantibodies. SSA exhibited the highest positivity rate at 60.9% (143 cases), followed by SSB at 36.6% (86 cases), ANuA at 24.3% (57 cases), U1-snRNP at 21.7% (51 cases), AHA at 15.3% (36 cases), CENP B at 14.0% (33 cases), ARPA at 11.9% (28 cases), Sm at 9.4% (22 cases), Scl-70 at 2.6% (6 cases), PM-Scl at 2.1% (5 cases), and Jo-1 at 0.4% (1 case). PCNA was not detected in any ANA-positive patient. In contrast, among the 28 ANA-negative BFP patients, only SSA and Scl-70 were positive, with positivity rates of 25.0% (7 cases) and 7.1% (2 cases), respectively, while all other autoantibodies tested were negative.

**Table 4:** Distribution of immune indicators stratified by ANA status in the false-positive group.

Items	ANA (+) (N = 235)		ANA (-) (N = 28)	
	N	(%)	N	(%)
U1-snRNP	51	21.7%	0	0.0%
Sm	22	9.4%	0	0.0%
SS-A	143	60.9%	7	25.0%
SS-B	86	36.6%	0	0.0%
Scl-70	6	2.6%	2	7.1%
PM-Scl	5	2.1%	0	0.0%
Jo-1	1	0.4%	0	0.0%
CENP B	33	14.0%	0	0.0%
PCNA	0	0.0%	0	0.0%
ANuA	57	24.3%	0	0.0%
AHA	36	15.3%	0	0.0%
ARPA	28	11.9%	0	0.0%

### 3.5 Proposed diagnostic algorithm for BFP reactions

**Figure 2:**
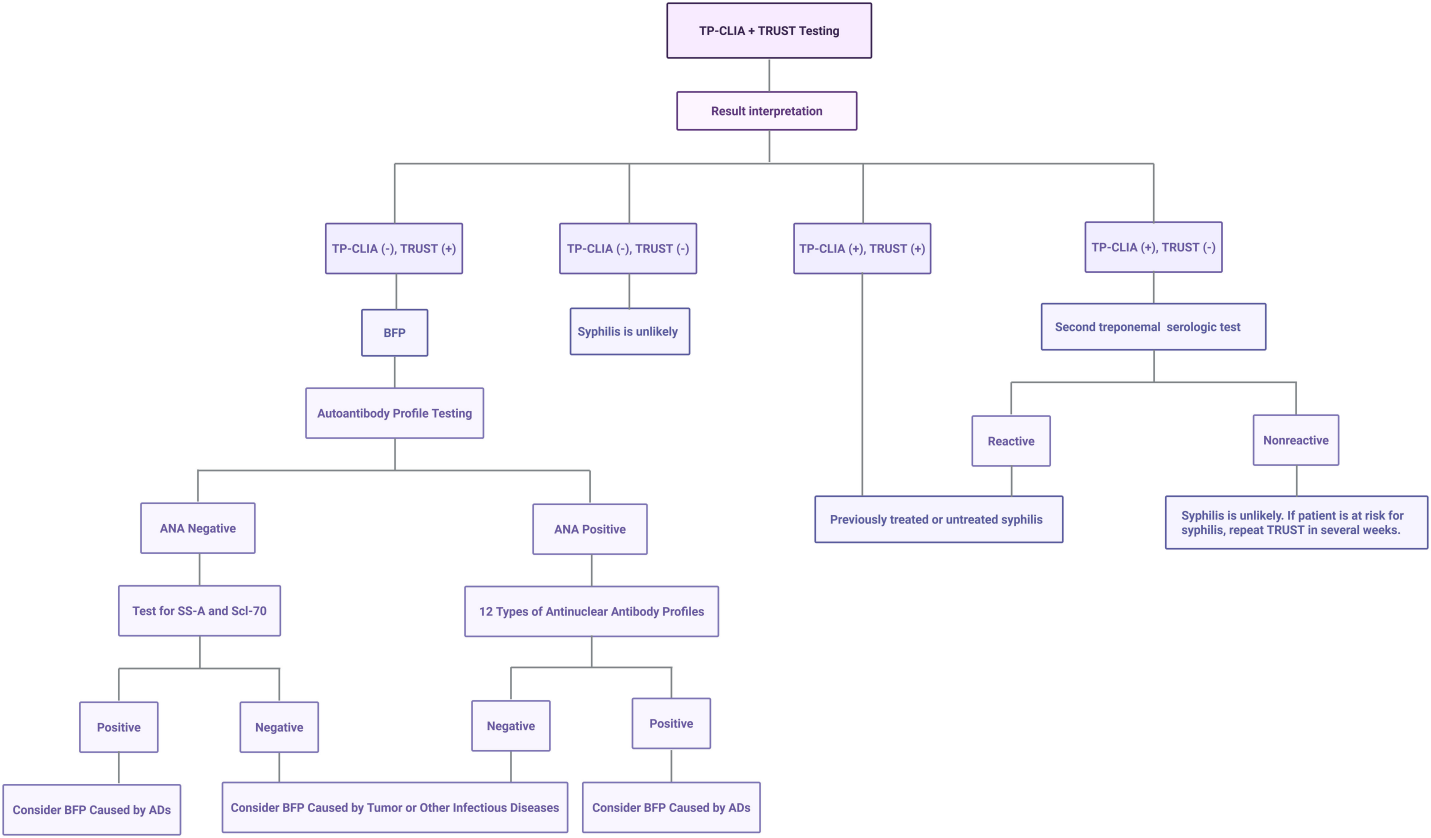
Diagnostic algorithm for BFP reactions in syphilis serological testin.g.

Based on the findings of this study, we propose a laboratory diagnostic algorithm for BFP reactions (**Figure 2**). The algorithm is initiated when a patient presents with TP-CLIA-negative and TRUST-positive results in the absence of syphilis history or symptoms. Upon entry into the BFP diagnostic pathway, autoantibody profiling is performed stratified by ANA status. For ANA-positive patients, a comprehensive panel including U1-snRNP, Sm, SS-A, SS-B, Scl-70, PM-Scl, Jo-1, CENP B, PCNA, ANuA, AHA, and ARPA is recommended. For ANA-negative patients, testing focuses on SS-A and Scl-70 based on our finding that these were the only autoantibodies detectable in this subgroup. Positive autoantibody results suggest that the BFP reaction is attributable to autoimmune diseases, whereas negative results prompt consideration of malignancies or infections. It is important to note that a negative ANA result should not be used as the sole basis for excluding an autoimmune etiology, as some SLE patients may test ANA-negative (Azhary et al., 2025).

## 4 Discussion

This study systematically evaluated the disease spectrum, demographic characteristics, and autoantibody profiles associated with BFP reactions in syphilis serological testing. In the analysis of 87 patients with false-positive reactions, the disease spectrum was broad, encompassing malignancies, autoimmune diseases, infectious diseases, cardiovascular diseases, and neurological disorders, among which cases of unknown etiology accounted for the highest proportion at 34.5%. A critical question arises regarding these unexplained cases: could they represent entirely healthy individuals? However, the reported incidence of BFP reactions in the general population is extremely low, approximately 0.59%, and less than 1 in 4,000 in rigorously screened healthy subjects (Moore et al., 1940; Smikle et al., 1990). Therefore, it is highly unlikely that all 30 BFP cases (34.5%) in our retrospective cohort represent completely healthy individuals. A more plausible interpretation is that these patients may have been in a subclinical disease state—such as early-stage or atypical autoimmune diseases, occult infections, or other undiagnosed conditions—or that the lack of a definitive diagnosis reflects incomplete clinical data due to outpatient settings with limited follow-up. Previous studies have also demonstrated that a proportion of apparently healthy individuals with BFP reactions eventually develop CTD upon long-term follow-up, further supporting the subclinical disease hypothesis (Knight and Wilkinson, 1963). This finding suggests that false-positive reactions may occur not only in patients with known autoimmune or infectious diseases, but also in those with undiagnosed or subclinical conditions, underscoring the critical need for enhanced follow-up and comprehensive evaluation in such patients. It is precisely this subset of patients with undiagnosed conditions that highlights the importance of in-depth research into BFP reactions and their underlying etiologies. Notably, most patients in our study (86.2%) had low TRUST titers (≤1:4). Consistent with our findings, Ishihara et al. also reported a substantial proportion of unexplained cases (44%) among patients with BFP reactions, with 85.9% presenting titers ≤1:4 (Ishihara et al., 2021).

In this cross-sectional cohort of 1068 cases, the incidence of BFP reactions differed significantly across disease groups. Patients with ADs had the highest BFP rate (52.3%), with rates of 75.9%, 62.4%, and 52.3% in RA, CTD, and SS, respectively, whereas that in SLE was 33.0%. The disparity in BFP rates across different ADs stems fundamentally from variations in the types and properties of autoantibodies. These autoantibodies can cross-react with the cardiolipin antigen used in NTTs (e.g., TRUST), leading to false-positive results (Rivera-Correa and Rodriguez, 2018; Chen et al., 2026). Notably, although SLE patients exhibited a relatively lower BFP rate, their TRUST titers showed a broader distribution (ranging from 1:1 to 1:4), suggesting that a subset of SLE patients may have enhanced nonspecific immune activation. In the present study, among EBV antibody-positive patients, BFP reactions were observed only in the 1–19 age group, and all such cases were IgM-positive, suggesting that acute EBV infection may be associated with heterophilic antibodies (Luo et al., 2025). In contrast, chronic or past infections are less likely to cause BFP. The two EBV antibody-positive patients in this age group were diagnosed with infectious mononucleosis (IM) and bronchopneumonia, respectively. This finding is consistent with the study by Matsuura et al., who also reported significantly elevated nontreponemal antibody titers in patients with EBV-associated IM (Matsuura, 2025; Matsuura et al., 2025). Among patients with malignancies, the BFP rate was 36.1%, with all titers ≤1:2, suggesting that tumor-related immune dysregulation may lead to low-titer BFP reactions through non-specific B cell activation or the induction of antiphospholipid antibodies (aPL) (Tincani et al., 2010). Among LAC-positive patients, the BFP rate was 8.5%, with a female predominance (85.7%), which is associated with the high expression of aPL in patients with APS or SLE. aPL may cross-react with the cardiolipin antigen used in nontreponemal syphilis tests, providing a potential molecular mechanism for BFP (Schultz, 1997).

To our knowledge, this is the first study to propose a systematic diagnostic algorithm specifically tailored for BFP reactions in syphilis serological testing that integrates ANA stratification with targeted autoantibody profiling. Previous algorithms have focused primarily on the traditional versus reverse screening sequences for syphilis diagnosis (Papp et al., 2024), but none have provided a disease-specific, autoantibody-guided pathway for BFP workup. The proposed algorithm addresses this gap by offering clinicians a structured, evidence-based approach to navigate the differential diagnosis of BFP reactions. The proposed algorithm has several practical utilities. First, it reduces unnecessary diagnostic testing in ANA-negative patients by limiting autoantibody screening to SS-A and Scl-70, thereby conserving healthcare resources. Second, it provides clear clinical guidance for differentiating autoimmune-mediated BFP from other etiologies. However, several limitations should be acknowledged. The diagnostic algorithm proposed in this study is intended to provide clinicians with a probability-based diagnostic shortcut, rather than an absolute diagnostic conclusion. In this algorithm, when an ANA-negative patient is also SS-A- and Scl-70-negative, screening for neoplastic and infectious diseases is prioritized, as these etiologies have the highest relative prevalence in the specific serological context of this study. Furthermore, other non-autoimmune and non-infectious causes—such as liver disease, recent vaccination, and drug reactions—may rarely present as a BFP reaction. Therefore, this algorithm should be applied in conjunction with a comprehensive clinical evaluation, and any negative result in the laboratory workup should prompt clinical follow-up rather than directly terminating the diagnostic process. Additionally, the algorithm is derived from a single-center cohort and requires external validation in diverse populations.

This study has several notable strengths. To our knowledge, it is the first to conduct a comprehensive evaluation of the disease spectrum and autoantibody profiles associated with BFP reactions in syphilis serological testing using two complementary sampling approaches—a retrospective cohort and a large cross-sectional cohort of over 1,000 patients. Unlike previous single-disease reports, our parallel comparison across multiple disease categories (autoimmune diseases, EBV infection, malignancies, and LAC positivity) enables direct risk stratification and provides a hierarchical understanding of BFP etiology. Furthermore, by stratifying autoantibody profiles by ANA status in BFP patients, we offer novel quantitative evidence to guide tiered diagnostic testing.

This study has several limitations. First, the retrospective cases relied on medical record data, which may be subject to information bias. Second, although the cross-sectional sample covered a wide range of diseases, the sample sizes of some subgroups were relatively small, limiting the feasibility of multivariate analysis. Furthermore, the dynamic changes of BFP reactions and their association with disease activity have not been systematically evaluated. Future prospective cohort studies are warranted to further explore the immunological mechanisms underlying BFP reactions and their potential value in disease monitoring.

## 5 Conclusion

BFP reactions in serological tests for syphilis are most frequently observed in patients with ADs, particularly SLE, SS, CTD, and RA, and typically present with low TRUST titers (≤1:2). Notably, BFP reactions can also occur in malignancies and acute EBV infection, with the latter being age-restricted to children and adolescents. Autoantibody profiling demonstrates that ANA-positive BFP patients exhibit a broad, distinct autoantibody repertoire, whereas ANA-negative patients show limited positivity, primarily to SS-A and Scl-70. These findings indicate that BFP reactions are not a homogeneous phenomenon but rather reflect diverse underlying immunological mechanisms that vary with the disease context. Comprehensive clinical evaluation integrating autoantibody testing, TRUST titer assessment, age, sex, and underlying disease status is essential to accurately identify BFP reactions, avoid misdiagnosis of syphilis, and prevent unnecessary antibiotic treatment.

## Data Availability

The original contributions presented in the study are included in the article/supplementary material. Further inquiries can be directed to the corresponding author.
